# Alleviation of the Adverse Effect of Dietary Carbohydrate by Supplementation of *Myo*-Inositol to the Diet of Nile Tilapia (*Oreochromis niloticus*)

**DOI:** 10.3390/ani10112190

**Published:** 2020-11-23

**Authors:** Jiahua Zhu, Jingyu Pan, Xiaodan Wang, Yuxing Huang, Chuanjie Qin, Fang Qiao, Jianguang Qin, Liqiao Chen

**Affiliations:** 1 Laboratory of Aquaculture Nutrition and Environmental Health, School of Life Sciences, East China Normal University, Shanghai 200241, China; 51181300037@stu.ecnu.edu.cn (J.Z.); 51191300021@stu.ecnu.edu.cn (J.P.); 10171900135@stu.ecnu.edu.cn (Y.H.); fqiao@bio.ecnu.edu.cn (F.Q.); 2 Key Laboratory of Sichuan Province for Fishes Conservation and Utilization in the Upper Reaches of the Yangtze River, Neijiang Normal University, Neijiang 641100, China; qinchuanjie@njtc.edu.cn; 3 College of Science and Engineering, Flinders University, Adelaide 5001, Australia; jian.qin@flinders.edu.au

**Keywords:** carbohydrate, lipid synthesis, lipid catabolism, *myo*-inositol, Nile tilapia

## Abstract

**Simple Summary:**

Recently, the price escalation of fishmeal has made aquaculture nutritionists to consider using carbohydrate in aquafeed to spare the use of dietary protein. However, the high carbohydrate diet could induce lipid metabolism disorder, impair antioxidant capacity, reduce nonspecific immunity and decrease resistance to a pathogen in farmed fish. *Myo*-inositol is regarded as a vitamin-like essential nutrient for most aquatic animals. Previous studies have shown that dietary supplementation with *myo*-inositol can reduce lipid accumulation in tissues and decrease the chance of becoming a fatty liver. To explore the mechanism of *myo*-inositol on alleviating the adverse effect of the high carbohydrate diet in Nile tilapia, six diets contained either low carbohydrate (30%) or high carbohydrate (45%) with three levels of *myo*-inositol supplementation (0, 400 and 1200 mg/kg diet) to each level of the carbohydrate diet. After an 8-week trial, the result showed that additive *myo*-inositol in the diet could significantly improve the growth performance and increase the crude protein content of fish. The addition of *myo*-inositol could effectively decrease the lipid accumulation induced by the high carbohydrate diet by accelerating the transportation of cholesterol back to the liver and promoting the lipid decomposition.

**Abstract:**

This study investigated the effect of dietary *myo*-inositol (MI) on alleviating the adverse effect of the high carbohydrate diet in Nile tilapia (*Oreochromis niloticus*). Six diets contained either low carbohydrate (LC 30%) or high carbohydrate (HC 45%) with three levels of MI supplementation (0, 400 and 1200 mg/kg diet) to each level of the carbohydrate diet. After an 8-week trial, the fish fed 400 mg/kg MI under HC levels had the highest weight gain and fatness, but the fish fed 1200 mg/kg MI had the lowest hepatosomatic index, visceral index and crude lipid in the HC group. The diet of 1200 mg/kg MI significantly decreased triglyceride content in the serum and liver compared with those fed the MI supplemented diets regardless of carbohydrate levels. Dietary MI decreased triglyceride accumulation in the liver irrespective of carbohydrate levels. The content of malondialdehyde decreased with increasing dietary MI at both carbohydrate levels. Fish fed 1200 mg/kg MI had the highest glutathione peroxidase, superoxide dismutase, aspartate aminotransferase and glutamic-pyruvic transaminase activities. The HC diet increased the mRNA expression of key genes involved in lipid synthesis (*DGAT, SREBP, FAS*) in the fish fed the diet without MI supplementation. Dietary MI significantly under expressed fatty acid synthetase in fish fed the HC diets. Moreover, the mRNA expression of genes related to lipid catabolism (*CPT, ATGL, PPAR-α*) was significantly up-regulated with the increase of dietary MI levels despite dietary carbohydrate levels. The gene expressions of gluconeogenesis, glycolysis and MI biosynthesis were significantly down-regulated, while the expression of the pentose phosphate pathway was up-regulated with the increase of MI levels. This study indicates that HC diets can interrupt normal lipid metabolism and tend to form a fatty liver in fish. Dietary MI supplement can alleviate lipid accumulation in the liver by diverging some glucose metabolism into the pentose phosphate pathway and enhance the antioxidant capacity in *O. niloticus*.

## 1. Introduction

Recently, the price escalation of fishmeal has made aquaculture nutritionists to consider using carbohydrate in aquafeed to spare the use of dietary protein [1,2]. Teleosts are known to be glucose intolerant with slow serum glucose clearance and hyperglycemia after a high intake of carbohydrate (HC) [3]. Nevertheless, the use of an appropriate level of carbohydrate as an alternative source of energy can improve oxidative protection in common dentex (*Dentex dentex*) juveniles [4]. The HC diet could induce lipid metabolism disorder, impair antioxidant capacity, reduce nonspecific immunity and decrease resistance to a pathogen in farmed fish [1,5,6]. Therefore, the diet with HC had been consistently linked to the high risk of hypertriglyceridemia, obesity, type 2 diabetes mellitus and fatty liver disease in fish [5]. Although the HC diet has been widely used in aquaculture, little attention has been paid to the negative effect of dietary HC in fish. With the increasing interest of using more carbohydrate in aquaculture diets, it is necessary to investigate the method to mitigate the negative effects of dietary carbohydrate on fish.

The structure of *myo*-inositol (MI) is similar to glucose, and it is a biologically active isomer of inositol in cell membranes [7,8]. The MI is the structural base for some secondary messengers, and it is also involved in lipid signaling, osmolarity, glucose and insulin metabolism in land animals [9,10]. In mammals, dietary supplementation with MI can effectively ameliorate certain endocrine diseases such as diabetes and insulin resistance as MI is closely related to carbohydrate metabolism [11]. Due to the de novo synthesis pathway, free MI could be de novo synthesized with glucose-6-phosphate (G6P), which is catalyzed by *myo*-inositol-1-phosphate synthase (*MIPS*) and *myo*-inositol monophosphatase (*IMPA1*) [12,13,14]. At the same time, G6P is involved in the pathways of carbohydrate metabolism [8,11]. Therefore, the *myo*-inositol biosynthesis (MIB) pathway is associated with carbohydrate metabolism and dietary supplementation with MI can also regulate lipid metabolism. The dietary MI can reduce the accumulation of triglycerides (TG) and decrease the expression of lipogenic genes and the activity of lipogenic proteinsin in rats with a nonalcoholic fatty liver [15]. In turbot *Scophthalmus maximus*, MI plays a vital role in transmembrane signal transfer, protection of the liver, and lipid metabolism [16]. In other aquatic animals, dietary supplementation with MI can reduce lipid accumulation in tissues and decrease the chance of becoming a fatty liver [17]. Although MI can reduce lipid accumulation in aquatic animals, the underlying molecular mechanism is still not clear.

The *Oreochromis niloticus* is an excellent farmed fish, which is promoted by Food and Agriculture Organization of the United Nations (FAO) due to its fast growth, high yield potential, low oxygen tolerance, euryhaline habitat, disease resistance and high fecundity [18,19]. Therefore, *O. niloticus* is an excellent model species for studying carbohydrate metabolism and lipid metabolism. The objective of this study was to investigate the effect of dietary *myo*-inositol on alleviating the adverse effects of high carbohydrate diets in *O. niloticus*.

## 2. Materials and Methods

### 2.1. Diet Preparation and Experimental Fish

Six semi-purified diets were prepared with a 2 × 2 factorial design are shown in Table 1. The basal diet contained 38% crude protein and 7% crude lipid. Corn starch was used as the source of carbohydrate. The feed used was the base purified feed. In this experiment, six different diets were prepared with two carbohydrate levels: low carbohydrate (LC 30%) and high carbohydrate (HC 45%) and three levels of MI supplementation (0, 400 and 1200 mg/kg diet) at each level of the carbohydrate diet. The diets were extruded into 2 mm pellets, air-dried and then stored at −20 °C until use.

Nile tilapia (*O. niloticus*) used in this experiment were obtained from a commercial fish hatchery in Guangdong Province (Guangdong Tianfa Fish Fry Development Co. LTD, Guangzhou, China). Fish were transported to the Biological Experimental Station of East China Normal University. During the acclimation period, the fish were fed with apparent satiety hand-fed twice daily by using a commercial diet. The water temperature was maintained at 27 ± 1 °C. After the two-week acclimation, the fish were fasted for 24 h prior to the experiment. A total of 540 juvenile Nile tilapias (1.45 ± 0.5 g) were selected and randomly distributed into eighteen 200-L tanks with 30 fish per tank. During the eight-week trial, all fish were hand-fed twice daily at 08:30 and 17:30 at a daily ration of 4% body weight. During the feeding trial, the environmental condition was maintained at 27 ± 1 °C, 5.0-6.0 mg L^−1^ dissolved oxygen, 7.3–7.6 pH, and a period of 12 h light and 12 h dark.

### 2.2. Sample Collection and Chemical Analysis

At the end of the trial, before fish were weighed and sampled, we stopped feeding for 24 h. The fish were weighed by tank, and the number of fish was counted to determine weight gain (WG) and survival (SR). Twelve fish of each treatment (four per tank) were euthanized (MS-222 at 20 mg/L) (tricaine methanesulfonate, Western Chemicals, Inc., Ferndale, WA, USA) and blood was rapidly collected from the caudal vein with a 1 mL syringe (Klmediacal, Haimen, China) and centrifuged for serum preparation (4,500 rpm, 10 min and 4 °C). The serum was immediately frozen at −80 °C for further analysis. Then the body length, viscera and liver weight of each fish were measured to calculate viscerosomatic index (VIS), hepatosomatic index (HSI) and condition factor (CF) respectively. The liver and muscle were collected for biochemical and molecular assays. The liver tissue was fixed in 4% paraformaldehyde for histological analysis. After that, 12 fish were collected in each treatment group and temporarily stored at −20 °C for the analysis of fish body composition.

### 2.3. Methods of Measurement

#### 2.3.1. Growth Performance and Body Composition

Weight gain (WG %) = 100 × (final body weight—initial body weight)/initial body weight;

Survival rate (SR %) = 100 × (final fish number/initial fish number); 

Feed conversion ratio (FCR) = total feed intake weight/(final body weight—initial body weight); 

Condition factor (CF %) = 100 × wet body weight/body length; 

Hepatosomatic index (HSI %) = 100 × wet hepatopancreas weight/wet body weight; 

Visceral index (VIS %) = 100 × wet visceral weight/wet body weight [13,17].

#### 2.3.2. Proximate Composition

Proximate composition of the whole body was determined by the standard methods (AOAC, 135 1995). Moisture was determined by gravimetric analysis following oven-drying at 105 °C. Crude protein and total lipid were determined by the Kjeldahl method (KjeltecTM 8200, Foss, Sweden) and the chloroform/methanol method, respectively.

#### 2.3.3. Histological Analysis

Three fish livers per tank were fixed in 4% paraformaldehyde solution for 48 h, washed in 70% ethanol solution, and then transferred to a 70% ethanol solution for storage until histological analysis. The paraffin production process, image collection and sample measurement of were determined according to the methods in previous studies, and digital images were taken using Image-Pro plus 6.0 [20,21,22].

#### 2.3.4. Biochemical Indicators

The contents of glucose (F006-1-1), triglyceride (TG, A110-1-1), high-density lipoprotein (HDL-C, A112-1-1), low-density lipoprotein (LDL-C, A113-1-1) and total cholesterol (T-CHO, A111-1-1) in the serum were all determined using the corresponding commercial kits (Nanjing Jiancheng Bioengineering Institute, Nanjing, China). The relevant steps were carried out according to the instructions. The total protein (A045-4-2) in the liver was also determined using the commercial kit (Nanjing Jiancheng Bioengineering Institute, Nanjing, China) according to the manufacturer’s guidelines. Serum insulin levels were analyzed by the ELISA kit following the manufacturer’s protocol (Shanghai Enzyme-linked Biotechnology Co., Ltd., Shanghai, China). The liver tissue was weighed and mixed with saline water at the ratio of 1:9 by weight (pH = 7.4). Ice bath homogenization was performed, and the homogenate was centrifuged at 4500 rotations/min at 4 °C for 10 min. The supernatant was pipetted and put on ice for the test. The liver TG (A110-1-1) content and the activities of superoxide dismutase (SOD, A001-3-2), glutathione peroxidase (GSH-Px, A005-1-2), alkaline phosphatase (AKP, A059-2-2), acid phosphatase (ACP, A060-2-1), aspartate aminotransferase (AST/GOT, C010-2-1), glutamic-pyruvic transaminase (ALT/GPT, C009-2-1) and muscle glycogen (A043-1-1) were determined using the corresponding commercial kits (Nanjing Jiancheng Bioengineering Institute, Nanjing, China).

#### 2.3.5. Gene Expression Analysis

Primers designed based on the *O. niloticus* transcriptome genome sequences are presented in Table 2. The primer amplification efficiency of all genes was between 90% and 110%. Total RNA was extracted by TRIzol^®^ reagent (RN0101, Invitrogen, Shanghai, China). The quantity and concentration of total RNA were measured by the Nanodrop 2000 (Thermo Fisher Scientific, Wilmington, NC, USA). The first-strand cDNA synthesis was performed using the PrimeScriptTM RT Reagent kit (KR116, Tiangen Biotech, Beijing, China).

The reaction volume used in qRT-PCR was 20 μL containing 10 μL 2 × Ultra SYBR Mixture (CWbio, Nanjing, China), 1.6 μL of each forward and reverse primers (2.5 μmol/μL), 1 μL of diluted cDNA (200 ng/μL) and 7.4 μL of RNAase free water. All procedures are performed according to the manufacturer’s instruction. *β-actin* was used as the reference gene. qRT-PCR data were analyzed with the 2^−ΔΔCt^ method.

### 2.4. Statistical Analysis

All statistical analyses were performed using SPSS Statistics 19.0 software. Normality and homoscedasticity assumptions were checked prior to the statistical analysis. Two-factor analysis of variance (ANOVA) was used to detect the two main factors of carbohydrate level and MI supplementation and their interaction. If significant interactions were detected, post hoc tests were used to assess the dependencies between the six treatments. All data are on average ± standard error (means ± SE) said. An asterisk (*) represents a significant difference of *p* < 0.05 between different MI levels in the same carbohydrate group. Double asterisks (**) represent a significant difference of *p* < 0.01 between different MI levels within the same carbohydrate group. A, B, C and a, b, c Values on bars without a common superscript letter are significantly different (*p* < 0.05), (a/A indicated the lowest value).

### 2.5. Ethical Statement

This research has been approved by Animal Ethics Committee of East China Normal University in February 2019 (permit number: E20120101).

## 3. Results

### 3.1. Growth Performance and Morphometric Parameters

Growth performance and morphometric parameters of *O. niloticus* fed different diets are showed in Table 3. No significant difference was observed in WG, SR, FCR and CF between carbohydrate levels (*p* > 0.05), but there were significant differences in these parameters between MI concentrations (*p* < 0.05). HSI and VIS were influenced by the interaction between carbohydrate levels and MI concentrations (*p* < 0.05). The highest WG value was found in the fish fed 400 mg/kg MI supplementation in the HC group (*p* < 0.05). No significant difference was observed in SR among the treatment groups. FCR was significantly decreased with the increase of dietary MI supplementation regardless of dietary carbohydrate levels (*p* < 0.05). The highest CF was found in the fish fed 400 mg/kg MI supplementation in the HC group (*p* < 0.05). HSI significantly decreased with the increase of MI supplementation in the HC group (*p* < 0.05). The lowest VIS was found in those fish fed 1200 mg/kg MI supplementation regardless of dietary carbohydrate levels (*p* < 0.05).

### 3.2. Whole-Body Proximate Composition

Crude lipid and moisture were affected by the interaction between carbohydrate levels and MI concentrations (*p* < 0.05). Crude protein was affected by the MI concentrations (*p* < 0.05). Crude lipid significantly decreased with the increase of MI supplementation regardless of dietary carbohydrate levels (*p* < 0.05). The highest moisture and crude protein occurred in the fish fed 400 mg/kg MI supplementation in the LC level (*p* < 0.05) (Table 4).

### 3.3. Parameters of Glycogen Content in Serum, Liver and Muscle

As shown in Table 5, serum glucose, serum insulin, liver glycogen and muscle glycogen contents were not affected by the interaction between carbohydrate levels and MI concentrations, or by carbohydrate levels (*p* > 0.05). No significant difference was found in serum glucose and muscle glycogen contents among the treatment groups (*p* > 0.05). Fish that were not fed MI supplementation showed a lower content of serum insulin (*p* < 0.05). The lower content of liver glycogen was found in the groups without MI supplementation regardless of dietary carbohydrate levels (*p* < 0.05).

### 3.4. Histology and Vacuolization of the Cytoplasm Area in the Liver

**Figure 1:**
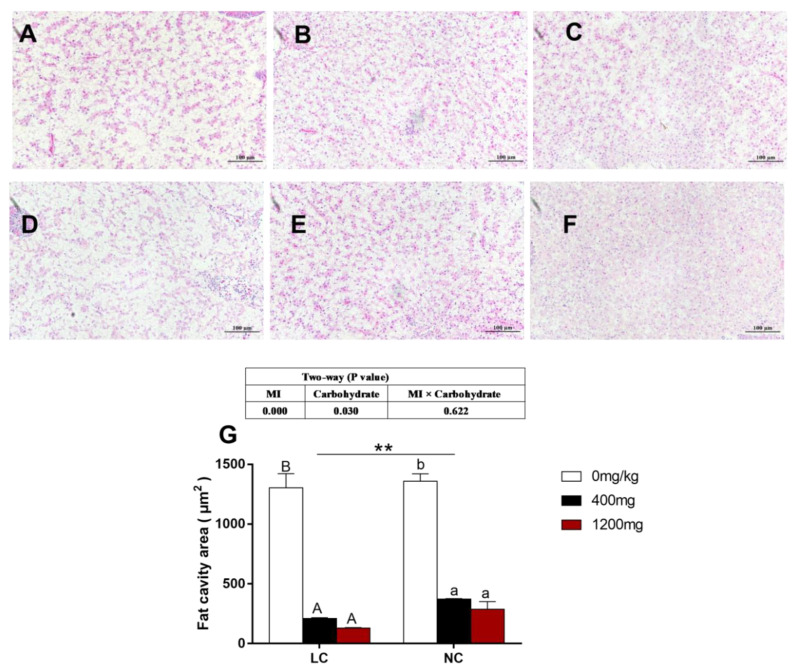
Histological characteristics of liver (40×). (**A**) LC-0 mg/kg; (**B**) LC-400 mg/kg; (**C**) LC-1200 mg/kg; (**D**) HC-0 mg/kg; (**E**) HC-400 mg/kg; (**F**) HC-1200 mg/kg; (**G**) lipid droplet area of the section area. Double asterisks (**) represent a significant difference of *p* < 0.01 between same level of MI groups. a, b and A, B Values on bars without a common superscript letter are significantly different (*p* < 0.05).

After 8 weeks of the feeding trial, the liver morphology of *O. niloticus* fed 400 mg/kg and 1200 mg/kg MI supplementation was normal, and showed fewer vacuoles in the cytoplasm compared with the fish fed 0 mg/kg MI supplementation regardless of dietary carbohydrate levels (Figure 1A–F). The addition of 400mg/kg and 1200 mg/kg MI significantly decreased the number of vacuoles in the cytoplasm of liver cells regardless of dietary carbohydrate levels (*p* < 0.05, Figure 1G).

### 3.5. The Expression of Genes Related to Lipid Metabolism

**Figure 2:**
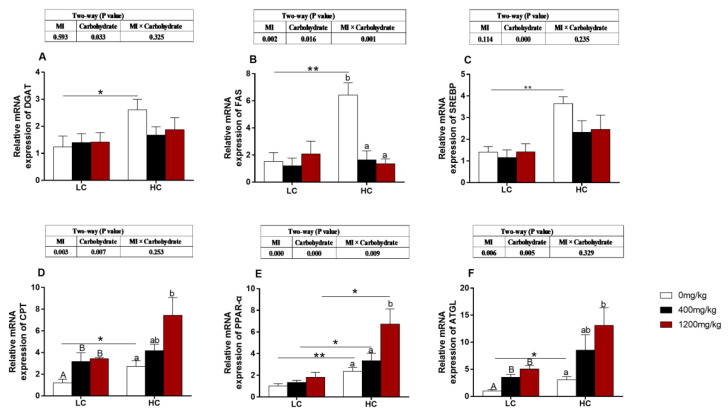
Effects of *myo*-inositol at different carbohydrate levels on mRNA levels of genes involved in lipid metabolism parameters in the liver of *O. niloticus*. Values are means (*n* = 3 replicate tanks) with standard errors represented by vertical bars. Asterisk (*) represents a significant difference of *p* < 0.05 between same level of MI groups. Double asterisks (**) represent a significant difference of *p* < 0.01 between same level of MI groups. a, b, c and A, B Values on bars without a common superscript letter are significantly different (*p* < 0.05) (a/A indicated the lowest value). (**A**) *DGAT2*: diacyltransferase; (**B**) *FAS*: fatty acid synthetase; (**C**) *SREBP*: sterol regulatory element binding protein; (**D**) *CPT*: carnitine palmityl transferase; (**E**) *PPAR-α*: peroxisome proliferator activated receptor-α; (**F**) *ATGL*: triglyceride lipase.

The expressions of *FAS* and *PPAR-α* genes in the liver were significantly affected by carbohydrate levels, MI concentrations and their interaction (*p* < 0.05, Figure 2B,E). The expressions of *DGAT, SREBP, CPT, FAS, PPAR-α* and *ATGL* were significantly affected by carbohydrate levels, and the expressions of *CPT, FAS, PPAR-α* and *ATGL* were also significantly affected by MI concentrations (*p* < 0.05, Figure 2A,C,D,F). Under 0 mg/kg MI supplementation, the expressions of *DGAT, FAS, SREBP, CPT* and *ATGL* were higher in the HC group than those in the LC level (*p* < 0.05, Figure 2A–D,F). No significant difference was observed in *DGAT*, and *SREBP* expressions in fish fed different levels of MI supplementation (*p* > 0.05, Figure 2A,C). The highest *FAS* gene expression level was found in the 0 mg/kg MI supplementation HC group (*p* < 0.05, Figure 2B). The expression levels of lipid-decomposition-related genes (*CPT, ATGL, PPAR-α*) were significantly up-regulated with the increase of MI supplementation regardless of dietary carbohydrate levels (*p* < 0.05, Figure 2D–F). The *PPAR-α* expression level was affected by carbohydrate levels, and the expression in the HC group was significantly higher than that in LC group with the same amount of MI addition (*p* < 0.05, Figure 2E).

### 3.6. The Expression of Carbohydrate-Metabolism-Related Genes

**Figure 3:**
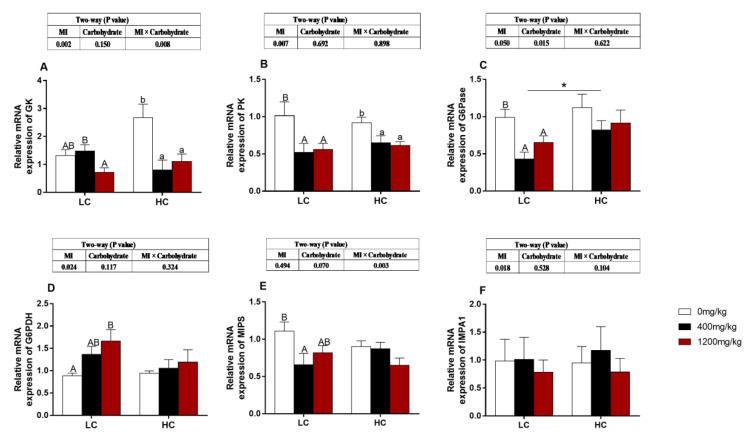
Effects of *myo*-inositol at different carbohydrate levels on mRNA levels of genes involved in glucose metabolism in the liver of *O. niloticus*. Values are means (*n* = 3 replicate tanks) with standard errors represented by vertical bars. Asterisk (*) represents a significant difference of *p* < 0.05 between same level of MI groups. a, b and A, B Values on bars without a common superscript letter are significantly different (*p* < 0.05) (a/A indicated the lowest value). (**A**) *GK*: hexokinase; (**B**) *PK*: pyruvate kinase; (**C**) *G6Pase*: glucose-6-phosphatase; (**D**) *G6PDH*: glucose-6-phosphate dehydrogenase; (**E**) *MIPS*: *myo*-inositol-1-phosphate synthase; (**F**) *IMPA1*: *myo*-inositol monophosphatase.

The expressions of *GK* and *MIPS* genes were affected by the interaction between carbohydrate levels and MI concentrations (*p* < 0.05, Figure 3A,E). The level of gene expression of *G6Pase* was only affected by carbohydrate levels (*p* < 0.05, Figure 3C). The expressions of *GK, PK, G6Pase* and *MIPS* were significantly down-regulated with increasing dietary MI supplementation regardless of dietary carbohydrate levels (*p* < 0.05, Figure 3A–C,E). The expression level of *G6Pase* was significantly up-regulated in the HC group when the MI concentration in the dietary was 400 mg/kg (*p* < 0.05, Figure 3C). The gene expression of *G6PDH* was significantly up-regulated with increasing dietary MI levels in the LC group (*p* < 0.05, Figure 3D). There was no significant difference in the expression of *IMPA1* in fish fed different diets (*p* > 0.05, Figure 3F).

### 3.7. Serum Lipid Contents and Liver TG Content Parameters

The contents of serum HDL-C and liver TG were significantly affected by the interaction between carbohydrate levels and MI concentrations (*p* < 0.05, Table 6). The contents of serum TG, serum HDL-C and liver TG were significantly affected by MI concentrations (*p* < 0.05). The content of serum HDL-C was also influenced by the carbohydrate level (*p* < 0.05). The significantly lower serum TG and liver TG contents were detected with increasing dietary MI supplementation regardless of dietary carbohydrate levels (*p* < 0.05). The highest serum HDL-C content was found in the group of 1200 mg/kg MI supplementation in the HC diet, and the contents of serum HDL-C in the groups of 400 mg/kg and 1200 mg/kg MI supplementation in the HC diet were higher than that in the LC diet (*p* < 0.05). No significant difference was found in serum LDL-C when fish were fed with different diets (*p* > 0.05). Fish fed the HC diet without MI supplementation showed the lowest content of serum T-CHO (*p* < 0.05).

### 3.8. Immune-Related and Antioxidative Parameters

**Figure 4:**
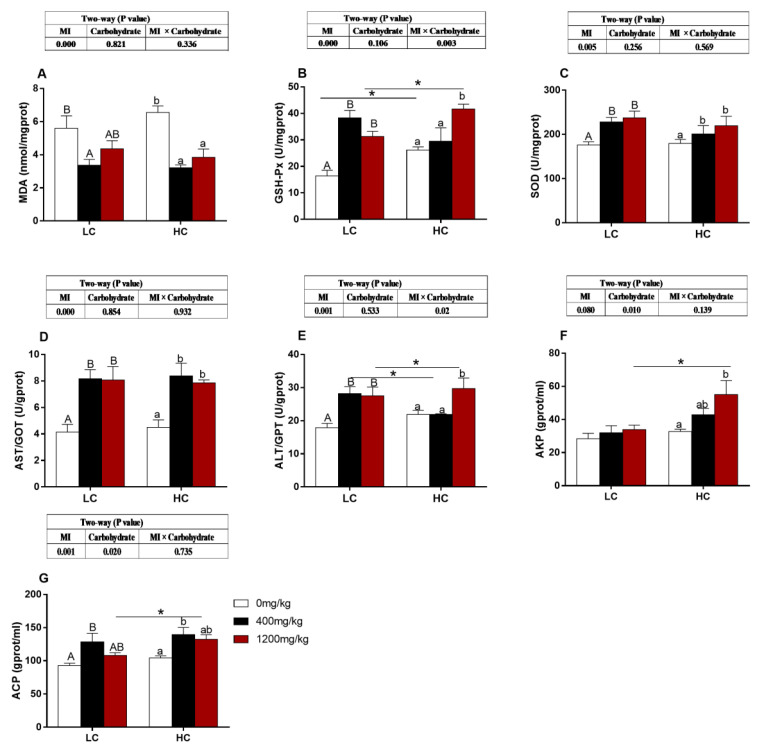
Effects of *myo*-inositol at different carbohydrate levels on immune-related parameters in the liver of *O. niloticus*. Values are means (*n* = 3 replicate tanks) with standard errors represented by vertical bars. Asterisk (*) represents a significant difference of *p* < 0.05 between same level of MI groups. a, b and A, B Values on bars without a common superscript letter are significantly different (*p* < 0.05) (a/A indicated the lowest value). (**A**) MDA: malonaldehyde. (**B**) GSH-Px: glutathione peroxidase; (**C**) SOD: superoxide dismutase; (**D**) AST/GOT: spartate aminotransferase; (**E**) ALT/GPT: glutamic-pyruvic transaminase; (**F**) AKP: alkaline phosphatase; (**G**) ACP: Acid phosphatas.

The activities of GSH-Px and ALT/GPT were significantly influenced by carbohydrate levels, MI concentrations and their interactions (*p* < 0.05, Figure 4B,E). The activity of AKP was significantly influenced by carbohydrate levels (*p* < 0.05, Figure 4F). The content of MDA was significantly decreased with increasing dietary MI supplementation regardless of dietary carbohydrate levels (*p* < 0.05, Figure 4A). The activities of GSH-Px, SOD, AST/GOT, ALT/GPT and ACP were significantly increased with increasing dietary MI supplementation regardless of dietary carbohydrate levels (*p* < 0.05, Figure 4B–E,G). The activity of AKP was significantly increased with increasing dietary MI supplementation in the HC diet (*p* < 0.05, Figure 4F).

## 4. Discussion

In the present study, the HC diets significantly affected growth, immunity, carbohydrate metabolism, lipid metabolism, and the health of liver tissue, which are similar to those observed in the blunt snout bream (*Megalobrama amblycephala*), Nile tilapia (*Oreochromis niloticus*) and European seabass (*Dicentrarchus labrax*) fed HC diets [5,23,24]. In the present study, the HC feed increased the weight gain and HSI, but dietary MI supplementation decreased HSI. This result may be due to the reason that HC diets can induce the synthesis of lipid from excess glucose in the liver [25,26,27]. Some studies have suggested that dietary MI deficiency can cause high accumulation of triacylglycerol, cholesterol, and non-esterified lipids in the mammalian liver, indicating that MI plays a crucial role in lipid metabolism [11,28]. As expected, the fish crude lipid, vacuolization of the cytoplasm and TG content in the liver were decreased with the increase of dietary MI. The MI is a precursor of inositol phosphates and is a vital second messenger signaling molecule in cellular processes, such as lipid signaling, glucose, and insulin metabolism [11,29]. The results in the current study suggest that MI can affect the lipid synthesis and metabolism in the body, thereby relieving lipid accumulation in body [16,30,31]. The qPCR results showed that dietary MI supplementation could down-regulate the expression of genes related with lipid synthesis (*FAS*) and up-regulate the expression of genes (*CPT, PPAR-α, ATGL*) related with lipid metabolism. The *FAS* plays a key role in the opposite process of de novo lipogenesis by converting acetyl-CoA and malonyl-CoA into fat [32,33].

*PPAR-α* has been identified as a critical regulator for hepatic lipid metabolism to control the transcription of genes involved in fatty acids beta-oxidation, lipoprotein metabolism, glucose metabolism, hepatic inflammation, and hepatocyte peroxisome proliferation [34]. *CPT* is the main regulatory enzyme in mitochondrial fatty acid oxidation because it is the catalyzing enzyme of the reaction from fatty acyl-CoAs into fatty acylcarnitines [35,36]. *ATGL* is a critical lipolysis lipase, and the lack or low expression of *ATGL* would result in a defect of lipolysis and the accumulation of triacylglycerols in tissues [37,38,39,40,41]. So, the results of the present study showed that MI could alleviate lipid accumulation by promoting lipid decomposition and inhibiting lipid synthesis. A previous study has suggested that MI can regulate lipid metabolism by mediating insulin resistance [42]. 

The decomposition of liver glycogen accelerated with the addition of MI regardless of carbohydrate levels. This may be due to that MI could promote the decomposition of liver glycogen into glucose and increase the glucose content by increasing protein kinase B (PKB)/Akt phosphorylation, increasing the sensitivity of insulin and promoting the utilization of glucose [43,44,45,46,47]. However, the specific molecular mechanism remains to be confirmed in future studies. The G6P is an essential intermediate in carbohydrate metabolism. Most G6P comes from the glycolysis process catalyzed by *GK* or *HK*, and continues the glycolysis reaction by the catalysis of *PK*. A part of G6P would act as the substrate and enters the MIB pathway by the catalysis of *MIPS* and *IMPA1.* The other part of G6P enters the pentose phosphate pathway by the catalysis of *G6PDH* [48,49,50,51]. In the present study, the HC diet promoted glycolysis and gluconeogenesis, but these processes decreased with the addition of MI in the feed. Simultaneously, the addition of MI promotes the activities of glucose in the pentose phosphate pathway [52,53,54]. In the current study, MI might change glucose to the pentose phosphate pathway. NADPH is mostly generated by the pentose phosphate pathway being one of the main intracellular reducing agents and an essential co-factor required for the normal function of antioxidant cycles such as the glutathione thioredoxin systems [49,55,56,57]. In the present study, when a large amount of lipid accumulated in the body and caused lipid peroxidation, the addition of MI can promote more glucose to enter the pentose phosphate pathway to increase the ratio of NADPH/NADP^+^, which would increase the activity of the antioxidant system and maintain the cell health [56,58]. However, the *MIPS* and *IMPA1* tend to decrease with the addition of MI regardless of the carbohydrates level. It may be due to the feedback regulation in the body, and the addition of MI in the feed meets the normal needs of the body, and there is no need to synthesize excessive MI [8,59]. Therefore, the addition of MI to the HC diet can promote the decomposition of liver glycogen into glucose, and then promote glucose metabolism into pentose phosphate pathway, thus providing a large amount of energy for the body and reducing power to alleviate the oxidative damage caused by HC diet.

The contents of lipidemia can reflect whole-body lipid metabolism state was detected [60,61]. The TG is the product of one glycerol molecule and three fatty acid molecules esterification. Under a normal condition, the serum TG content maintains the dynamic balance, but a large amount of TG would accumulate when lipid metabolism was disturbed [62]. The contents of T-CHO, HDL-C and LDL-C in the serum can reflect lipid metabolism and transport capacity of the liver [62,63,64]. Physiologically, HDL-C is good cholesterol due to having an anti-atherogenic effect, because HDL-C is responsible for transporting CHO from extrahepatic tissues to liver for metabolism to prevent free CHO deposition in blood [60,65,66]. The results showed that the addition of MI in the HC diet can improve the transportation of CHO from blood and other peripheral tissues to the liver, which effectively improved the absorption and transportation of CHO on the HC diet in *O. niloticus* [60]. Therefore, the reason why dietary MI decreased the lipid accumulation might be that additional MI accelerated the transportation of CHO back to the liver and promoted the lipolysis reaction. 

The results of the present study demonstrate that the HC diet caused lipid deposition, which triggered lipid peroxidation and oxidative stress and then induced liver function damage. Similar findings have been found in hybrid grouper (*Epinephelus fuscoguttatus ♀ × E. lanceolatus ♂*), Surubim (*Pseudoplatystoma reticulatum × P corruscans*) and *Oreochromis niloticus* [1,5,67]. The accumulation of large amounts of lipid will damage the liver structure, physiological function and lipid metabolism disorder, which would lead to excessive free radicals and finally cause oxidative damage to the body [68,69]. SOD is a vital antioxidant enzyme and can eliminate excess free radicals, reduce and inhibit lipid peroxidation, and protect cells from oxidative damage [70,71]. GSH-Px is a vital peroxidase widely existing in the body, which can protect the structure and function of cell membranes from interference and damage of oxides because it can reduce the toxic peroxides to nontoxic hydroxyl compounds [72,73,74]. In the present study, the SOD and GSH-Px activities were significantly improved with the increase of MI supplementation regardless of dietary carbohydrate levels. At the same time, the MDA content was significantly decreased with the increase of MI supplementation regardless of dietary carbohydrate levels. This may be because that dietary MI decreased the accumulation of lipid in the liver which was easy to cause oxidative stress, so the antioxidant capacity of the body had a corresponding increase [75,76]. Moreover, the activities of GST/GOT and ALT/GPT were the most sensitive indicators of liver cell damage, which is increased by dietary MI, this indicates that the addition of MI alleviates the damage of liver cells and avoids the formation of fatty liver [77]. AKP and ACP are essential enzymes for growth metabolism, homeostasis and health [17,78,79]. Therefore, the activities of AKP and ACP in the liver tissue also indirectly reflect the health of the liver [80]. The activities of AKP and ACP were consistent with the results of antioxidant-related enzymes in the present study. These results further demonstrate that dietary MI can help avoid oxidative stress caused by lipid peroxidation, increase the antioxidant capacity and thus maintain the normal structure and function of the liver cells [11,15].

## 5. Conclusions

The HC diet could cause the accumulation of lipid in the liver of *Oreochromis niloticus*, destroy the physiological function and structure of the liver, form fatty liver, and finally affect fish growth and survival. In the present study, additive MI in the diet could significantly improve the growth performance increase the crude protein content and decreased the crude lipid content of fish. The addition of MI could effectively decrease the lipid accumulation induced by the HC diet by accelerating the transportation of CHO back to the liver and promoting the lipid decomposition. Moreover, supplemented MI also changed the glucose metabolism and promoted the activities of the pentose phosphate pathway in the liver to produce more amount of NADPH, which could help enhance the antioxidant capacity of the liver to prevent it from oxidative stress caused by HC diet.

## Figures and Tables

**Table 1:** Formulation and chemical composition of experimental diets (g/kg dry basis).

Ingredients	Content (g/kg Dry Basis)					
Casein (Vitamin-Free)	320	320	320	320	320	320
Gelatin	80	80	80	80	80	80
Soybean oil	70	70	70	70	70	70
Corn starch	300	450	300	450	300	450
*Myo*-inositol ^c^ (mg/kg diet)	0	0	0.4	0.4	1.2	1.2
Vitamin premix ^a^	5	5	5	5	5	5
Mineral premix ^b^	5	5	5	5	5	5
Ca(H_2_PO_4_)_2_	15	15	15	15	15	15
Carboxymethyl cellulose	25	25	25	25	25	25
Cellulose	175.75	27.75	175.35	27.35	176.55	26.55
Phagostimulant	2	2	2	2	2	2
BHT	0.25	0.25	0.25	0.25	0.25	0.25
Total	1000	1000	1000	1000	1000	1000
Proximate composition						
Moisture	100.5	105.6	100.3	102.3	106.8	103.9
Crude protein	372.2	379.8	375.5	377.4	376.5	378.4
Total lipid	69.5	69.6	68.3	68.7	68.5	69.3
Ash	28.82	28.76	28.73	30.18	30.16	29.11

^a^ Vitamin premix (mg/kg diet): retinal palmitate (500,000 IU/g), 8; cholecalciferol (1,000,000 IU/g), 2; menadione, 10; DL-α-tocopherol acetate, 200; thiamin-HCl, 10; riboflavin, 12; pyridoxine-HCl, 10; D-calcium pantothenate, 32; amine nicotinic acid, 80; folic acid, 2; cyanocobalamin, 0.01; biotin, 0.2; choline chloride, 400; ascorbic acid, 60; α-cellulose, 4173.79; ^b^ Mineral premix (mg/kg diet): ZnSO_4_·H_2_O, 150; FeSO_4_·H_2_O, 40; MnSO_4_·H_2_O, 15.3; CuSO_4_·5H_2_O, 8.3; potassium iodide, 5; CoCl_2_·6H_2_O, 0.05; Na_2_SeO_3_, 0.09; α-cellulose, 4785.76; ^c^ Sangong Biotech, Ltd., Shanghai, China.

**Table 2:** Primer pair sequences and product size of the genes used for real-time PCR (qPCR).

Gene	Position	Primer Sequence	Length	Tm	Product Size (bp)
*GK*	Forward	GTCATCAACCTGATGCGGGA	20	60.18	163
	Reverse	ACCTGTCACGGAAACATGGG	20	59.75	
*PK*	Forward	GCTAACCAAGACTGGCAGGT	20	59.96	438
	Reverse	TGGAGGGATTCGTGGAGTCT	20	59.96	
*G6Pase*	Forward	GGATGCTAATGGGCCTGGTC	20	59.78	169
	Reverse	CAGCTACCAGTGTGCCTGTAA	21	59.60	
*G6PDH*	Forward	TCCAGAACCTCATGGTGCTT	20	60.18	312
	Reverse	GGCTCCTTGAAGGTAAGGACG	21	59.69	
*MIPS*	Forward	CGTCCTACGAGGGAACCTCT	20	60.39	179
	Reverse	GCAGAGTCTTTGCACGGAATA	21	58.65	
*IMPA1*	Forward	ATAAGCCGGGAAGCAGTCTC	20	59.53	132
	Reverse	GTGTTTGGTCGTTCGATGGTG	21	60.07	
*CPT*	Forward	GTGGGCGTCCAACTATGTCA	20	59.04	251
	Reverse	TACGCTCGTATTGGGCTGAG	20	60.12	
*PPAR-α*	Forward	GGGCCATAGTGTGAGTGTGA	20	59.75	245
	Reverse	TGGGTGTCCACCATGTCTAC	20	59.78	
*ATGL*	Forward	AAAACGTCCTGGTGACCCCAGT	21	59.98	104
	Reverse	TAGGAGGAATGATGCCACAGTACA	24	60.03	
*FAS*	Forward	ACAGCTGCAGACCCAGAATC	20	60.04	307
	Reverse	GTAGAAGGCAGAGGCTGCAA	20	60.04	
*DGAT2*	Forward	AGAGGAGCTGTAAGCTCGGA	20	60.03	157
	Reverse	AGTGCCTTTGAGGAATCCCG	20	60.04	
*SREBP*	Forward	ATGTCCCCATGTTCCCACTG	20	59.67	137
	Reverse	GCTAACGCATATGCCTCCCA	20	60.25	
*β-actin*	Forward	GGATTCACTCTGAGCGCCG	19	58.43	203
	Reverse	CCGTCTCCTTACCTTTGGGTG	21	59.12	

*GK*: hexokinase; *PK*: pyruvate kinase; *G6Pase*: glucose-6-phosphatase; *G6PDH*: glucose-6-phosphate dehydrogenase; *MIPS*: *myo*-inositol-1-phosphate synthase; *IMPA1*: *myo*-inositol monophosphatase; *CPT*: carnitine palmityl transferase; *PPAR-α*: peroxisome proliferator activated receptor-α; *ATGL*: triglyceride lipase; *FAS*: fatty acid synthetase; *DGAT2*: diacyltransferase; *SREBP*: sterol regulatory element binding protein.

**Table 3:** Growth performance and physiological parameters of *O. niloticus* fed different experiment diets.

Diets	WG (%)	SR (%)	FCR	CF (%)	HSI (%)	VIS (%)
LC-0	722.45 ± 6.21	91.11 ± 1.11	1.13 ± 0.54 ^B^	2.96 ± 4.67	1.76 ± 7.05	10.84 ± 18.04 ^A^
LC-400	739.35 ± 19.11	95.56 ± 2.94	1.09 ± 0.35 ^A^	3.06 ± 8.06	1.73 ± 10.35	12.98 ± 51.66 ^B^
LC-1200	766.75 ± 48.54	92.22 ± 2.22	1.09 ± 1.56 ^A,B^	3.09 ± 6.52	1.61 ± 10.22	11.75 ± 39.55 ^A^
HC-0	753.32 ± 42.11 ^a^	94.44 ± 2.94	1.10 ± 1.81 ^b^	2.96 ± 5.76 ^a^	2.08 ± 11.26 ^b,^*	11.07 ± 21.41 ^b^
HC-400	867.73 ± 43.46 ^b^	88.33 ± 5.00	1.12 ± 3.09 ^b^	3.14 ± 6.01 ^b^	1.55 ± 10.10 ^a^	11.30 ± 25.83 ^b^
HC-1200	777.85 ± 9.53 ^a^	97.78 ± 1.11	0.96 ± 0.42 ^a^	3.03 ± 6.40 ^a,b^	1.76 ±9.91 ^a^	9.92 ± 23.63 ^a^
AN0VA (P)						
MI	0.140	0.486	0.120	0.073	0.012	0.002
carbohydrates	0.042	0.795	0.029	0.940	0.223	0.001
MI × carbohydrates	0.143	0.073	0.081	0.506	0.043	0.017

Data were expressed as mean ± SEM (standard error of the mean) (*n* = 6). Values in the same line with different superscripts are significantly different (*p* < 0.05). Values are means (*n* = 3 replicate tanks) with standard errors represented by vertical bars. Asterisk (*) represents a significant difference of *p* < 0.05 between same level of MI groups. a, b and A, B Values on bars without a common superscript letter are significantly different (*p* < 0.05).

**Table 4:** Proximate composition of *O. niloticus* (% wet weight) fed different experiment diets.

Diets	Moisture (%)	Crude Lipid (%)	Crude Protein (%)
LC-0	74.06 ± 0.11 ^A^	13.87 ± 0.77 ^B^	45.72 ± 0.19 ^A^
LC-400	76.70 ± 0.47 ^B^	11.90 ± 0.91 ^A^	54.77 ± 2.61 ^B,^**
LC-1200	73.62 ± 0.56 ^A^	15.62 ± 0.53 ^A,B^	45.26 ± 0.69 ^A^
HC-0	74.65 ± 0.21	15.80 ± 0.37 ^b^	43.77 ± 0.82
HC-400	74.39 ± 0.37	15.00 ± 0.46 ^b^	44.07 ± 0.39
HC-1200	74.65 ± 0.43	13.28 ± 0.18 ^a^	44.14 ± 1.02
AN0VA (P)			
MI	0.003	0.109	0.013
carbohydrates	0.488	0.133	0.068
MI × carbohydrates	0.006	0.003	0.076

Data were expressed as mean ± SEM (standard error of the mean) (*n* = 3). Values in the same line with different superscripts are significantly different (*p* < 0.05). Values are means (*n* = 3 replicate tanks) with standard errors represented by vertical bars. Double asterisks (**) represent a significant difference of *p* < 0.01 between same level of MI groups. a, b and A, B Values on bars without a common superscript letter are significantly different (*p* < 0.05).

**Table 5:** Serum, liver and muscle carbohydrate content parameters of *O. niloticus* fed different experiment diets.

Diets	Serum Glucose	Serum INS	Liver Glycogen	Muscle Glycogen
LC-0	4.23 ± 0.18	70.35 ± 1.56 ^A^	17.68 ± 0.92 ^B^	1.42 ± 0.16
LC-400	4.69 ± 0.31	85.75 ± 2.61 ^C^	17.66 ± 2.48 ^B^	1.44 ± 0.27
LC-1200	4.89 ± 0.22	80.01 ± 0.49 ^B^	14.07 ± 0.96 ^A^	1.46 ± 0.15
HC-0	5.44 ± 0.43	73.53 ± 0.81 ^a^	16.90 ± 0.51 ^b^	1.56 ± 0.18
HC-400	5.19 ± 0.32	83.87 ± 2.72 ^b^	14.57 ± 1.32 ^a^	1.67 ± 0.20
HC-1200	5.68 ± 0.22	80.07 ± 1.68 ^b^	14.58 ± 0.87 ^a^	1.58 ± 0.12
AN0VA (P)				
MI	0.119	0.000	0.000	0.924
carbohydrates	0.000	0.767	0.154	0.507
MI × carbohydrates	0.418	0.405	0.077	0.650

Data were expressed as mean ± SEM (standard error of the mean) (*n* = 3). Values in the same line with different superscripts are significantly different (*p* < 0.05). Values are means (*n* = 3 replicate tanks) with standard errors represented by vertical bars. a, b and A, B, C Values on bars without a common superscript letter are significantly different (*p* < 0.05). Serum INS: Serum insulin.

**Table 6:** Serum and liver lipid content parameters of *O. niloticus* fed different experiment diets.

Diets	Serum TG	Serum HDL-C	Serum LDL-C	Serum T-CHO	Liver TG
LC-0	2.05 ± 0.26 ^B^	1.05 ± 0.08	2.72 ± 0.26	2.16 ± 0.96	0.31 ± 0.02 ^B^
LC-400	1.77 ± 0.14 ^A,B^	0.96 ± 0.07	2.87 ± 0.19	2.18 ± 0.13	0.22 ± 0.33 ^A^
LC-1200	0.35 ± 0.12 ^A^	1.15 ± 0.07	2.82 ± 0.10	2.19 ± 0.12	0.21 ± 0.03 ^A^
HC-0	2.25 ± 0.09 ^b^	1.21 ± 0.04 ^a^	2.46 ± 0.12	1.90 ± 0.05 ^a^	0.29 ± 0.13 ^b^
HC-400	1.98 ± 0.20 ^b^	1.58 ± 0.09 ^b,^**	2.83 ± 0.21	2.47 ± 0.14 ^b^	0.20 ± 0.02 ^a^
HC-1200	1.31 ± 0.10 ^a^	2.02 ± 0.13 ^c,^**	2.93 ± 0.05	2.49 ± 0.11 ^b^	0.21 ± 0.02 ^a^
AN0VA (P)					
MI	0.000	0.000	0.202	0.052	0.000
carbohydrates	0.734	0.000	0.655	0.662	0.074
MI × carbohydrates	0.389	0.002	0.567	0.112	0.030

Data were expressed as mean ± SEM (standard error of the mean) (*n* = 3). Values in the same line with different superscripts are significantly different (*p* < 0.05). Values are means (*n* = 3 replicate tanks) with standard errors represented by vertical bars. Double asterisks (**) represent a significant difference of *p* < 0.01 between same level of MI groups. a, b, c and A, B Values on bars without a common superscript letter are significantly different (*p* < 0.05). TG: triglycerides; HDL-C: high-density lipoproteincholesterol; LDL-C: low-density lipoproteincholesterol; T-CHO: total cholesterol.

## References

[1] Li S., Wang A., Li Z., Zhang J., Sang C., Chen N. (2020). Antioxidant defenses and non-specific immunity at enzymatic and transcriptional levels in response to dietary carbohydrate in a typical carnivorous fish, hybrid grouper (*Epinephelus fuscoguttatus* female symbol × *E. lanceolatus* male symbol). Fish Shellfish Immun..

[2] Li S., Sang C., Wang A., Zhang J., Chen N. (2019). Effects of dietary carbohydrate sources on growth performance, glycogen accumulation, insulin signaling pathway and hepatic glucose metabolism in largemouth bass, *Micropterus salmoides*. Aquaculture.

[3] Liu D., Deng K., Sampath W., Gu Z., Pan M., Zhang Y., Zhang W., Mai K. (2019). Responses of glucosensing system to glucose in Japanese flounder *Paralichthys olivaceus* fed diets with different carbohydrate content. Comp. Biochem. Physiol. B Biochem. Mol. Biol..

[4] Perez-Jimenez A., Abellan E., Arizcun M., Cardenete G., Morales A.E., Hidalgo M.C. (2017). Dietary carbohydrates improve oxidative status of common dentex (*Dentex dentex*) juveniles, a carnivorous fish species. Comp. Biochem. Physiol. A Mol. Integr. Physiol..

[5] Luo Y., Hu C.-T., Qiao F., Wang X.-D., Qin J.G., Du Z.-Y., Chen L.-Q. (2020). Gemfibrozil improves lipid metabolism in Nile tilapia *Oreochromis niloticus* fed a high-carbohydrate diet through peroxisome proliferator activated receptor-α activation. Gen. Comp. Endocr..

[6] Xu C., Liu W.B., Remo S.C., Wang B.K., Shi H.J., Zhang L., Liu J.D., Li X.F. (2019). Feeding restriction alleviates high carbohydrate diet-induced oxidative stress and inflammation of *Megalobrama amblycephala* by activating the AMPK-SIRT1 pathway. Fish Shellfish Immun..

[7] Khosravi S., Lim S.-J., Rahimnejad S., Kim S.-S., Lee B.-J., Kim K.-W., Han H.-S., Lee K.-J. (2015). Dietary *myo*-inositol requirement of parrot fish, *Oplegnathus fasciatus*. Aquaculture.

[8] Cui W., Ma A., Wang X., Huang Z. (2020). *Myo*-inositol enhances the low-salinity tolerance of turbot (*Scophthalmus maximus*) by modulating cortisol synthesis. Biochem. Biophys. Res. Commun..

[9] Bathena S.P., Huang J., Epstein A.A., Gendelman H.E., Boska M.D., Alnouti Y. (2012). Rapid and reliable quantitation of amino acids and *myo*-inositol in mouse brain by high performance liquid chromatography and tandem mass spectrometry. J. Chromatogr. B Anal. Technol. Biomed. Life Sci..

[10] Villalba H., Shah K., Albekairi T.H., Sifat A.E., Vaidya B., Abbruscato T.J. (2018). Potential role of *myo*-inositol to improve ischemic stroke outcome in diabetic mouse. Brain Res..

[11] Gonzalez-Uarquin F., Rodehutscord M., Huber K. (2020). *Myo*-inositol: Its metabolism and potential implications for poultry nutrition-a review. Poult. Sci..

[12] Buccafusca R., Venditti C.P., Kenyon L.C., Johanson R.A., Van Bockstaele E., Ren J., Pagliardini S., Minarcik J., Golden J.A., Coady M.J. (2008). Characterization of the null murine sodium/*myo*-inositol cotransporter 1 (Smit1 or Slc5a3) phenotype: Myo-inositol rescue is independent of expression of its cognate mitochondrial ribosomal protein subunit 6 (Mrps6) gene and of phosphatidylinositol levels in neonatal brain. Mol. Genet. Metab..

[13] Peres H., Lim C., Klesius P.H. (2004). Growth, chemical composition and resistance to Streptococcus iniae challenge of juvenile Nile tilapia (*Oreochromis niloticus*) fed graded levels of dietary inositol. Aquaculture.

[14] Wang X., Kultz D. (2017). Osmolality/salinity-responsive enhancers (OSREs) control induction of osmoprotective genes in euryhaline fish. Proc. Natl. Acad. Sci. USA.

[15] Shimada M., Ichigo Y., Shirouchi B., Takashima S., Inagaki M., Nakagawa T., Hayakawa T. (2019). Treatment with *myo*-inositol attenuates binding of the carbohydrate-responsive element-binding protein to the ChREBP-beta and FASN genes in rat nonalcoholic fatty liver induced by high-fructose diet. Nutr. Res..

[16] Cui W., Ma A. (2020). Transcriptome analysis provides insights into the effects of myo-inositol on the turbot Scophthalmus maximus. Fish Shellfish Immun..

[17] Bu X., Lin Z., Liu S., Wang C., Wang N., Lei Y., Zhu J., Wang X., Qin J.G., Chen L. (2020). Effects of *myo*-inositol on growth performance, body composition, antioxidant status, non-specific immunity and lipid metabolism of juvenile Chinese mitten crab (*Eriocheir sinensis*). Aquacult. Nutr..

[18] Magouz F.I., Dawood M.A.O., Salem M.F.I., El-Ghandour M., Van Doan H., Mohamed A.A.I. (2020). The role of a digestive enhancer in improving the growth performance, digestive enzymes activity, and health condition of Nile tilapia (*Oreochromis niloticus*) reared under suboptimal temperature. Aquaculture.

[19] Han S.-L., Wang J., Li L.-Y., Lu D.-L., Chen L.-Q., Zhang M.-L., Du Z.-Y. (2020). The regulation of rapamycin on nutrient metabolism in Nile tilapia fed with high-energy diet. Aquaculture.

[20] Li M., Wang X., Qi C., Li E., Du Z., Qin J.G., Chen L. (2018). Metabolic response of Nile tilapia (*Oreochromis niloticus*) to acute and chronic hypoxia stress. Aquaculture.

[21] Schmitt V.H., Schmitt C., Hollemann D., Weinheimer O., Mamilos A., Kirkpatrick C.J., Brochhausen C. (2019). Tissue expansion of lung bronchi due to tissue processing for histology—A comparative analysis of paraffin versus frozen sections in a pig model. Pathol. Res. Pract..

[22] Yao J., Li Q., Zhou B., Wang D., Wu R. (2018). Advantages of infrared transflection micro spectroscopy and paraffin-embedded sample preparation for biological studies. Spectrochim. Acta Part A Mol. Biomol. Spectrosc..

[23] Yu C., Zhang J., Qin Q., Liu J., Xu J., Xu W. (2020). Berberine improved intestinal barrier function by modulating the intestinal microbiota in blunt snout bream (*Megalobrama amblycephala*) under dietary high-fat and high-carbohydrate stress. Fish Shellfish Immun..

[24] Machado M., Castro C., Oliva-Teles A., Costas B. (2019). Interactive effects of dietary vegetable oil and carbohydrate incorporation on the innate immune response of European seabass (*Dicentrarchus labrax*) juveniles subjected to acute stress. Aquaculture.

[25] Aparecida de Franca S., Pavani Dos Santos M., Nunes Queiroz da Costa R.V., Froelich M., Buzelle S.L., Chaves V.E., Giordani M.A., Pereira M.P., Colodel E.M., Marlise Balbinotti Andrade C. (2014). Low-protein, high-carbohydrate diet increases glucose uptake and fatty acid synthesis in brown adipose tissue of rats. Nutrition.

[26] Menezes A.L., Pereira M.P., Buzelle S.L., Dos Santos M.P., de Franca S.A., Baviera A.M., Andrade C.M., Garofalo M.A., Kettelhut Ido C., Chaves V.E. (2013). A low-protein, high-carbohydrate diet increases de novo fatty acid synthesis from glycerol and glycerokinase content in the liver of growing rats. Nutr. Res..

[27] Chen S., Zhuang Z., Yin P., Chen X., Zhang Y., Tian L., Niu J., Liu Y. (2019). Changes in growth performance, haematological parameters, hepatopancreas histopathology and antioxidant status of pacific white shrimp (*Litopenaeus vannamei*) fed oxidized fish oil: Regulation by dietary *myo*-inositol. Fish Shellfish Immun..

[28] L’Abbate S., Nicolini G., Forini F., Marchetti S., Di Lascio N., Faita F., Kusmic C. (2020). *Myo*-inositol and d-chiro-inositol oral supplementation ameliorate cardiac dysfunction and remodeling in a mouse model of diet-induced obesity. Pharmacol. Res..

[29] Blind R.D. (2020). Structural analyses of inositol phosphate second messengers bound to signaling effector proteins. Adv. Biol. Regul..

[30] Foster S.R., Dilworth L.L., Thompson R.K., Alexander-Lindo R.L., Omoruyi F.O. (2017). Effects of combined inositol hexakisphosphate and inositol supplement on antioxidant activity and metabolic enzymes in the liver of streptozotocin-induced type 2 diabetic rats. Chem. Biol. Interact..

[31] Lete M.G., Tripathi A., Chandran V., Bankaitis V.A., McDermott M.I. (2020). Lipid transfer proteins and instructive regulation of lipid kinase activities: Implications for inositol lipid signaling and disease. Adv. Biol. Regul..

[32] Ali A., Zhang Y., Fu M., Pei Y., Wu L., Wang R., Yang G. (2020). Cystathionine gamma-lyase/H2S system suppresses hepatic acetyl-CoA accumulation and nonalcoholic fatty liver disease in mice. Life Sci..

[33] Salie M.J., Thelen J.J. (2016). Regulation and structure of the heteromeric acetyl-CoA carboxylase. Biochim. Biophys. Acta..

[34] Nikroo H., Hosseini S.R.A., Fathi M., Sardar M.A., Khazaei M. (2020). The effect of aerobic, resistance, and combined training on PPAR-alpha, SIRT1 gene expression, and insulin resistance in high-fat diet-induced NAFLD male rats. Physiol. Behav..

[35] Liu Q., Liao Y., Wu Y., Xu M., Sun Z., Ye C. (2020). Cloning and characterization of carnitine palmitoyltransferase Iα (CPT1α) from obscure puffer (*Takifugu obscurus*), and its gene expression in response to different lipid sources. Aquacult. Rep..

[36] Shi X.C., Sun J., Yang Z., Li X.X., Ji H., Li Y., Chang Z.G., Du Z.Y., Chen L.Q. (2017). Molecular characterization and nutritional regulation of carnitine palmitoyltransferase (CPT) family in grass carp (*Ctenopharyngodon idellus*). Comp. Biochem. Physiol. B Biochem. Mol. Biol..

[37] Ayisi C.L., Yamei C., Zhao J.-L. (2018). Genes, transcription factors and enzymes involved in lipid metabolism in fin fish. Agri Gene.

[38] Yan X., Qin C., Deng D., Yang G., Feng J., Lu R., Wang G., Nie G. (2020). Regulation of glucose and lipid metabolism by insulin and glucagon in vivo and in vitro in common carp Cyprinus carpio L.. Aquacult. Rep..

[39] Lu R.-H., Jia S.-Z., Yang F., Qin C.-B., Zhang Y.-R., Meng X.-L., Yan X., Feng J.-C., Nie G.-X. (2020). The function of miR-122 in the lipid metabolism and immunity of grass carp (*Ctenopharyngodon idellus*). Aquacult. Rep..

[40] Wang T., Wei Q., Liang L., Tang X., Yao J., Lu Y., Qu Y., Chen Z., Xing G., Cao X. (2020). OSBPL2 Is Required for the Binding of COPB1 to ATGL and the Regulation of Lipid Droplet Lipolysis. iScience.

[41] Li S., Sang C., Zhang J., Li Z., Chen N. (2018). Molecular cloning, expression profiling of adipose triglyceride lipase (ATGL) and forkhead box O1 (FoxO1), and effects of dietary carbohydrate level on their expression in hybrid grouper (*Epinephelus fuscoguttatus* ♀ × *E. lanceolatus* ♂). Aquaculture.

[42] Genazzani A.D. (2016). Inositol as putative integrative treatment for PCOS. Reprod. Biomed. Online.

[43] Croze M.L., Soulage C.O. (2013). Potential role and therapeutic interests of *myo*-inositol in metabolic diseases. Biochimie.

[44] Vong C.T., Tseng H.H.L., Kwan Y.W., Lee S.M., Hoi M.P.M. (2019). G-protein coupled receptor 55 agonists increase insulin secretion through inositol trisphosphate-mediated calcium release in pancreatic beta-cells. Eur. J. Pharmacol..

[45] Croze M.L., Vella R.E., Pillon N.J., Soula H.A., Hadji L., Guichardant M., Soulage C.O. (2013). Chronic treatment with *myo*-inositol reduces white adipose tissue accretion and improves insulin sensitivity in female mice. J. Nutr. Biochem..

[46] Gu Z., Mu H., Shen H., Deng K., Liu D., Yang M., Zhang Y., Zhang W., Mai K. (2019). High level of dietary soybean oil affects the glucose and lipid metabolism in large yellow croaker *Larimichthys crocea* through the insulin-mediated PI3K/AKT signaling pathway. Comp. Biochem. Physiol. B Biochem. Mol. Biol..

[47] Kim J.N., Han S.N., Kim H.K. (2014). Phytic acid and myo-inositol support adipocyte differentiation and improve insulin sensitivity in 3T3-L1 cells. Nutr. Res..

[48] Osada-Oka M., Hashiba Y., Akiba S., Imaoka S., Sato T. (2010). Glucose is necessary for stabilization of hypoxia-inducible factor-1alpha under hypoxia: Contribution of the pentose phosphate pathway to this stabilization. FEBS Lett..

[49] Rodrigues J., Branco V., Lu J., Holmgren A., Carvalho C. (2015). Toxicological effects of thiomersal and ethylmercury: Inhibition of the thioredoxin system and NADP^(+)^-dependent dehydrogenases of the pentose phosphate pathway. Toxicol. Appl. Pharmacol..

[50] Ma A., Cui W., Wang X., Zhang W., Liu Z., Zhang J., Zhao T. (2020). Osmoregulation by the *myo*-inositol biosynthesis pathway in turbot *Scophthalmus maximus* and its regulation by anabolite and c-Myc. Comp. Biochem. Physiol. Part A Mol. Integr. Physiol..

[51] Olavarria V.H., Figueroa J.E., Mulero V. (2012). Prolactin-induced activation of phagocyte NADPH oxidase in the teleost fish *gilthead seabream* involves the phosphorylation of p47phox by protein kinase C. Dev. Comp. Immun..

[52] Hristov M., Landzhov B., Yakimova K. (2020). Cafeteria diet-induced obesity reduces leptin-stimulated NADPH-diaphorase reactivity in the hypothalamic arcuate nucleus of rats. Acta Histochem..

[53] Oliveira G.T., Rossi I.C., Kucharski L.C., Da Silva R.S. (2004). Hepatopancreas gluconeogenesis and glycogen content during fasting in crabs previously maintained on a high-protein or carbohydrate-rich diet. Comp. Biochem. Physiol. Part A Mol. Integr. Physiol..

[54] Liang X., Wang J., Gong G., Xue M., Dong Y., Wu X., Wang X., Chen C., Liang X., Qin Y. (2017). Gluconeogenesis during starvation and refeeding phase is affected by previous dietary carbohydrates levels and a glucose stimuli during early life in Siberian sturgeon (*Acipenser baerii*). Anim. Nutr..

[55] Gelman S.J., Naser F., Mahieu N.G., McKenzie L.D., Dunn G.P., Chheda M.G., Patti G.J. (2018). Consumption of NADPH for 2-HG Synthesis Increases Pentose Phosphate Pathway Flux and Sensitizes Cells to Oxidative Stress. Cell Rep..

[56] de Freitas-Silva L., Rodriguez-Ruiz M., Houmani H., da Silva L.C., Palma J.M., Corpas F.J. (2017). Glyphosate-induced oxidative stress in *Arabidopsis thaliana* affecting peroxisomal metabolism and triggers activity in the oxidative phase of the pentose phosphate pathway (OxPPP) involved in NADPH generation. J. Plant Physiol..

[57] Wasylenko T.M., Ahn W.S., Stephanopoulos G. (2015). The oxidative pentose phosphate pathway is the primary source of NADPH for lipid overproduction from glucose in *Yarrowia lipolytica*. Metab. Eng..

[58] Zhu Z., Umehara T., Tsujita N., Kawai T., Goto M., Cheng B., Zeng W., Shimada M. (2020). Itaconate regulates the glycolysis/pentose phosphate pathway transition to maintain boar sperm linear motility by regulating redox homeostasis. Free Radic. Biol. Med..

[59] Cui W., Ma A., Huang Z., Liu Z., Yang K., Zhang W. (2020). *myo*-inositol facilitates salinity tolerance by modulating multiple physiological functions in the turbot *Scophthalmus maximus*. Aquaculture.

[60] Fitria P.D., Amin M., Lokapirnasari W.P., Lamid M. (2020). Supplementation of fermented coffee-peel flour to increase high-density lipoprotein (HDL) cholesterol, docosahexaenoic acids (DHA) and eicosapentaenoic acids (EPA) deposition in tilapia fillet. Biocatal. Agric. Biotechnol..

[61] Zitnanova I., Oravec S., Janubova M., Konarikova K., Dvorakova M., Laubertova L., Kralova M., Simko M., Muchova J. (2020). Gender differences in LDL and HDL subfractions in atherogenic and nonatherogenic phenotypes. Clin. Biochem..

[62] Zhang X., Han Z., Zhong H., Yin Q., Xiao J., Wang F., Zhou Y., Luo Y. (2019). Regulation of triglyceride synthesis by estradiol in the livers of hybrid tilapia (*Oreochromis niloticus* female symbol × *O. aureus* male symbol). Comp. Biochem. Physiol. Part B Biochem. Mol. Biol..

[63] Xie S., Yin P., Tian L., Liu Y., Niu J. (2020). Lipid metabolism and plasma metabolomics of juvenile largemouth bass *Micropterus salmoides* were affected by dietary oxidized fish oil. Aquaculture.

[64] Liu M., Wallmon A., Wallin R., Saldeen T. (2003). Effects of stable fish oil and simvastatin on plasma lipoproteins in patients with hyperlipidemia. Nutr. Res..

[65] Zenimaru Y., Takahashi S., Takahashi M., Yamada K., Iwasaki T., Hattori H., Imagawa M., Ueno M., Suzuki J., Miyamori I. (2008). Glucose deprivation accelerates VLDL receptor-mediated TG-rich lipoprotein uptake by AMPK activation in skeletal muscle cells. Biochem. Biophys. Res. Commun..

[66] Kontush A. (2020). HDL and Reverse Remnant-Cholesterol Transport (RRT): Relevance to Cardiovascular Disease. Trends Mol. Med..

[67] Sousa L.C., Moromizato B.S., de Almeida V.D.N.S., Miasaki C.T., Takahashi L.S., Biller J.D. (2020). There is more than one way of feeding carnivorous fish: Surubim (*Pseudoplatystoma reticulatum* × *P. corruscans*) are able to cope with carbohydrates rich diets, but there is a trade-off between growth and immunity. Anim. Feed Sci. Technol..

[68] Kaushal N., Gupta M., Kulshreshtha E. (2020). Hempseed (*Cannabis sativa*) lipid fractions alleviate high-fat diet-induced fatty liver disease through regulation of inflammation and oxidative stress. Heliyon.

[69] Lakshmi S.P., Reddy A.T., Kodidhela L.D., Varadacharyulu N.C. (2020). Epigallocatechin gallate diminishes cigarette smoke-induced oxidative stress, lipid peroxidation, and inflammation in human bronchial epithelial cells. Life Sci..

[70] Xavier W.d.S., Leclercq E., Carvalho P.L.P.F., Vicente I.S.T., Guimarães M.G., Rodrigues E.J.D., Milanezi R.C., Barbé F., Sartori M.M.P., Pezzato L.E. (2020). The putative effect of a SOD-rich melon pulp-concentrate on growth performance and antioxidant status of Nile tilapia (*Oreochromis niloticus*) under heat/dissolved oxygen-induced stress. Aquaculture.

[71] Wang C., Liang Y., Fang Y., Chang X. (2019). Effects of cyclical short-term food deprivation and refeeding on compensatory growth and gene expression of SOD, GPX and HSP70 in *Schizothorax wangchiachii*. Fish Shellfish Immun..

[72] Shang X., Yu P., Yin Y., Zhang Y., Lu Y., Mao Q., Li Y. (2020). Effect of selenium-rich Bacillus subtilis against mercury-induced intestinal damage repair and oxidative stress in common carp. Comp. Biochem. Physiol. Part C Toxicol. Pharmacol..

[73] Sun J.L., Zhao L.L., Liao L., Tang X.H., Cui C., Liu Q., He K., Ma J.D., Jin L., Yan T. (2020). Interactive effect of thermal and hypoxia on largemouth bass (*Micropterus salmoides*) gill and liver: Aggravation of oxidative stress, inhibition of immunity and promotion of cell apoptosis. Fish Shellfish Immun..

[74] Abdel-Latif H.M.R., Soliman A.A., Sewilam H., Almeer R., Van Doan H., Alagawany M., Dawood M.A.O. (2020). The influence of raffinose on the growth performance, oxidative status, and immunity in Nile tilapia (*Oreochromis niloticus*). Aquacult. Rep..

[75] Yan S., Meng Z., Tian S., Teng M., Yan J., Jia M., Li R., Zhou Z., Zhu W. (2020). Neonicotinoid insecticides exposure cause amino acid metabolism disorders, lipid accumulation and oxidative stress in ICR mice. Chemosphere.

[76] Chen S.-J., Gan L., Guo Y.-C., Tian L.-X., Liu Y.-J. (2020). Changes in growth performance, aflatoxin B1 residues, immune response and antioxidant status of *Litopenaeus vannamei* fed with AFB1-contaminated diets and the regulating effect of dietary *myo*-inositol supplementation. Food Chem..

[77] Lin J.D., Lin P.Y., Chen L.M., Fang W.H., Lin L.P., Loh C.H. (2010). Serum glutamic-oxaloacetic transaminase (GOT) and glutamic-pyruvic transaminase (GPT) levels in children and adolescents with intellectual disabilities. Res. Dev. Disabil..

[78] Li S.A., Jiang W.D., Feng L., Liu Y., Wu P., Jiang J., Kuang S.Y., Tang L., Tang W.N., Zhang Y.A. (2018). Dietary *myo*-inositol deficiency decreased intestinal immune function related to NF-kappaB and TOR signaling in the intestine of young grass carp (*Ctenopharyngodon idella*). Fish Shellfish Immun..

[79] Chen S., Yu Y., Gao Y., Yin P., Tian L., Niu J., Liu Y. (2019). Exposure to acute ammonia stress influences survival, immune response and antioxidant status of pacific white shrimp (*Litopenaeus vannamei*) pretreated with diverse levels of inositol. Fish Shellfish Immun..

[80] Cai X., Zhang J., Lin L., Li Y., Liu X., Wang Z. (2020). Study of a noninvasive detection method for the high-temperature stress response of the large yellow croaker (*Larimichthys crocea*). Aquacult. Rep..

